# Meetings of Societies

**Published:** 1895-06

**Authors:** 


### MEETINGS OF SOCIETIES.

### Bristol Medico-Chirurgical Society.

*April 10th, 1895.*

Dr. A. J. Harrison, President, in the Chair.

The Hon. Secretary read a letter he had received from Mrs. Bernard, tendering her best thanks on behalf of the family of the late Dr. Davey for the kind sympathy expressed in the resolution passed at the meeting of the Bristol Medico-Chirurgical Society on March 13th.

Mr. C. E. S. Flemming showed a patient with an uncommon form of skin-disease, and a specimen of ulcerative endocarditis.

Dr. A. E. Aust Lawrence read a paper on backward displacement of the gravid uterus, and illustrated the communication with diagrams.

Dr. J. E. Shaw and Mr. J. Paul Bush read a paper on a case of tumour of the brain (see p. 99).

Mr. G. Munro Smith showed lantern-slides and specimens prepared by Dr. D. S. Davies and Dr. W. Dowson, of pneumococci found in the bladder in a case of cystitis following pneumonia (see p. 115).

Dr. D. S. Davies showed some interesting lantern-slides of photographs of patients suffering from small-pox; also micro-photographs of the bacillus of enteric fever, tetanus, and diphtheria.

---

*May 8th*, 1895.

Dr. A. J. Harrison, President, in the Chair.

Mr. C. A. Morton showed (1) a patient with congenital dislocation of one hip, in which there was no lateral curvature of the spine; (2) a male patient with syphilitic disease of the knee-joint and syphilitic disease of the breast; (3) a patient with Dupuytren's contraction of the palmar fascia; (4) a specimen of multiple polypi of the large intestine, associated with cancer in the sigmoid flexure and rectum.

Dr. J. Michell Clarke showed a large solid tumour of the ovary removed by Mr. Greig Smith.

Dr. W. L. Christie showed a temporal bone with necrosis of mastoid and caries of tympanic cavity, with a tuberculous mass attached to the dura mater of the squamous region.

Dr. B. M. H. Rogers showed a specimen of aortic disease and aortic aneurism from a child.

Dr. J. Michell Clarke read a paper on the treatment of gall-stones by olive oil.

Dr. R. Shingleton Smith gave a lantern demonstration of slides illustrating the phenomena of crisis and the effects of various antipyretics in cases of pneumonia. In endeavouring to answer the question, "Can we do anything to favour and to expedite the crisis of pneumonia?" he expressed the opinion that the so-called expectant and restorative method had proved itself to be altogether a delusive failure, and that we might well expect to obtain better results from a more vigorous antipyretic and perhaps also some specific treatment, the dangers of which are far less than those of the disease when left uncontrolled.

J. Paul Bush, *Hon. Sec.*

---

### Devon and Exeter Medico-Chirurgical Society.

*March 15th*, 1895.

Mr. G. H. Johnson, President, in the Chair.

Mr. J. Leslie Callaghan read a paper on rupture of the perineum.

Dr. C. N. Lovely reported the following cases of clinical interest: (1) Melanotic sarcoma of liver; (2) perforating gastric ulcer without premonitory symptoms; (3) infantile malnutrition treated with humanised milk.

*April 19th*, 1895.

Mr. J. D. Harris, Vice-President, in the Chair.

Mr. J. D. Harris showed a specimen of hæmatoporphyrinuria.

Dr. W. Gordon read a paper on the diagnosis and treatment of pulmonary abscess.

---

*May 17th*, 1895.

Mr. J. D. Harris, Vice-President, in the Chair.

Mr. A. C. Roper showed a specimen of colic intussusception.

Mr. J. D. Harris showed a specimen of intussusception.

Dr. W. Gordon showed a specimen (microscopical) of tubercular kidney.

Mr. H. Andrew showed a specimen of acute congestion of kidney after thrombosis of renal vein, occurring in a case of necrosis of femur.

Mr. J. D. Harris read a paper on gradual intestinal obstruction.

R. V. Solly, *Hon. Sec.*

